# Supply Considerations for Scaling Up Clean Cooking Fuels for Household Energy in Low‐ and Middle‐Income Countries

**DOI:** 10.1029/2019GH000208

**Published:** 2019-12-03

**Authors:** E. Puzzolo, H. Zerriffi, E. Carter, H. Clemens, H. Stokes, P. Jagger, J. Rosenthal, H. Petach

**Affiliations:** ^1^ Department of Public Health and Policy University of Liverpool Liverpool United Kingdom; ^2^ Global LPG Partnership New York USA; ^3^ University of British Columbia, Forest Resources Management Canada; ^4^ Colorado State University, Civil and Environmental Engineering USA; ^5^ Hivos The Hague The Netherlands; ^6^ Project Gaia, Inc. Gettysburg PA USA; ^7^ University of Michigan, School for Environment and Sustainability USA; ^8^ Fogarty International Center, NIH USA; ^9^ U.S. Agency for International Development Washington DC USA

**Keywords:** clean cooking, fuel supply, household air pollution, clean energy, clean fuels

## Abstract

Promoting access to clean household cooking energy is an important subject for policy making in low‐ and middle‐income countries, in light of urgent and global efforts to achieve universal energy access by 2030 (Sustainable Development Goal 7). In 2014, the World Health Organization issued “Guidelines for Indoor Air Quality: Household Fuel Combustion”, which recommended a shift to cleaner fuels rather than promotion of technologies that more efficiently combust solid fuels. This study fills an important gap in the literature on transitions to household use of clean cooking energy by reviewing supply chain considerations for clean fuel options in low‐ and middle‐income countries. For the purpose of this study, we consider electricity, liquefied petroleum gas (LPG), alcohol fuels, biogas, and compressed biomass pellets burned in high performing gasifier stoves to be clean fuel options. Each of the clean fuels reviewed in this study, as well as the supply of electricity, presents both constraints and opportunities for enhanced production, supply, delivery, and long‐term sustainability and scalability in resource‐poor settings. These options are reviewed and discussed together with policy and regulatory considerations to help in making these fuel and energy choices available and affordable. Our hope is that researchers, government officials and policy makers, and development agencies and investors will be aided by our comparative analysis of these clean household energy choices.

## Key Points

- Designing effective energy policies that support and accelerate transitions to clean cooking fuels is a top global priority
- Countries depend on a portfolio of fuels for cooking and should plan an optimal mix of clean fuels, both in the medium and long‐term
- Risk and sustainability of cookfuel supply chains depend on multiple factors that ultimately influence long‐term viability and scale

## 1 Introduction

Burning solid fuels (i.e., dung, crop residues, firewood, charcoal, and mineral coal) for cooking results in high household air pollution (HAP) exposures for more than one‐third of the global population (Bonjour et al., 2013) and contributes to ambient air pollution exposures (Chafe et al., 2014). Household air pollution causes an estimated 1.6–3.8 million premature deaths annually (IHME, 2018; WHO, 2018).

Over the past several decades, development efforts at various scales have focused on providing improved cooking technologies for lower emission combustion of traditional solid fuels. Emerging scientific evidence following these efforts in numerous countries, including Malawi (Mortimer et al., 2017), China (Snider et al., 2018), India (Aung et al., 2016), as well as several recent systematic reviews (Pope et al., 2017; Thomas et al., 2015) suggests that reductions in HAP levels attained by transitions to solid fuels burning in improved cookstoves, or even fan‐assisted gasifier stoves, have been insufficient to achieve air quality close to the World Health Organization (WHO) safe guideline levels in field conditions (Mortimer et al., 2017; Pope et al., 2017). To achieve health‐relevant reductions in air pollution, a transition to clean fuels is essential. We restrict the scope of this analysis to household fuels for cooking. Use of “household energy” and associated terms (e.g., “clean energy”, “clean fuels”, “household fuels”, etc.) in this work pertains to household cooking energy. End‐uses such as space heating and lighting are considered outside the scope of this analysis. We further define clean household fuels/energy sources for cooking to include liquefied petroleum gas (LPG), biogas, alcohol (both ethanol and methanol), compressed biomass pellets, and electricity (grid and photovoltaic).

Despite a growing evidence base on the public health benefits of transitions to clean fuels for household cooking, multiple barriers to scaling of clean energy transitions in low‐ and middle‐income countries (LMICs) exist. They include, economic viability, household adoption and uptake, and appropriate enabling environments including standards and regulatory frameworks. Some of these barriers have been discussed and analyzed in detail, such as adoption and sustained use of clean cooking technologies (Kowsari & Zerriffi, 2011; Lewis & Pattanayak, 2012; Puzzolo et al., 2016), while others, including clean fuel supply chains for low and middle‐income countries, have not. This analysis focuses on the supply considerations for LMICs planning a transition to clean household fuels.

This study addresses the question: What are the current challenges to and opportunities for supplying clean cooking fuels for household use at multiple scales in LMICs? In response to this framing question, information is synthesized from research and case studies in LMICs where household transitions to clean fuels are being documented. Much of the existing evidence base has focused on cooking fuels, motivating our focus on clean cooking in this study.

## 2 Methods

We analyze supply side considerations for clean household fuels using a logic framework developed to support household energy policy decisions associated with scaling‐up household energy transitions in low‐income and resource‐constrained settings (Puzzolo et al., 2016; Quinn et al., 2018; Rosenthal et al., 2017). The 'Logic Model' includes five dimensions that are interlinked and ultimately determine whether and which household fuels/energy sources are appropriate for promotion and use at scale. The five dimensions are:

1. institutional environment: the extent of supportive policies, regulations, standards and tax regimes for different household energy fuels/sources;
2. industry structure and services: fuel supply chain aspects from storage to distribution and safety practices associated with supply, as well as after‐sale services;
3. energy pricing and costing: end‐user prices for different fuels available in the market in terms of ongoing costs and start‐up cost for the initial equipment;
4. consumer demand: knowledge of different fuel options including availability, accessibility and reliability of supply for each option, and also considering rural vs urban access and the ability to afford and safely use the different fuels options;
5. user and community needs and perceptions: how different fuels are accepted and used in daily practice; how risks of use are viewed and influence user behavior in fuel choices and uptake; equity considerations including understanding heterogeneous impacts for women and marginalized groups.

Based on the model, this study focuses specifically on the supply chain aspects captured under industry structure and services (ii) and aspects related to the ‘enabling environment' under institutional environment (i) and energy pricing and costing (iii), as these areas represent the most significant data gaps in the literature.

The Logic Model is used to: (1) compare clean fuel options for low‐income and resource‐constrained settings; (2) identify current challenges and opportunities to scaling‐up diverse clean fuel options; and (3) synthesize current knowledge of factors influencing fuel supply sustainability and risk. The outcome of this effort is a comprehensive decision support system for existing clean household fuel options for LMIC settings for policy‐makers, government officials, civil society, the private sector and other key stakeholders.

This analysis leverages a portfolio of 11 case studies from Asia, Africa and Latin‐America countries that describe transitions to clean household fuels (Quinn et al., 2018) using the RE‐AIM (reach, effectiveness, adaptation, implementation, maintenance) Framework (Glasgow et al., 1999). In each case study, a clean fuel program that aims to expand household access to clean fuels at scale, was critically reviewed.

### 2.1 Clean Fuel Definition and Sources

As noted above, for this analysis clean household fuels/energy for cooking include LPG, biogas, alcohol (both ethanol and methanol), compressed biomass pellets, and electricity (grid and photovoltaic). Piped natural gas (PNG) is not included because the market scale‐up potential for resource‐poor and last mile settings is limited, due to the high investments in infrastructure required. However, we acknowledge that PNG is certainly a suitable solution in urban settings where consumer density is high and governments (e.g. India) have made investments to expand its use (Parikh, 2018).

Biomass pellets and other forms of compressed biomass are included in the list of clean household fuels because they have the potential to be burned cleanly in highly performing advanced stoves (sometimes termed Tier 4 stoves in the early development of cookstove standards). Examples of these low emission stoves include the Mimi Moto pellet stove and a small number of other options which have shown promise under laboratory and recent field testing (Champion & Grieshop, 2019). Other forms of biomass such as charcoal are classified as polluting fuels and are not incorporated in this analysis. Charcoal, is sometimes perceived as an improvement over other forms of biomass (e.g., dung or firewood) and has been particularly important in energy transitions in Sub‐Saharan Africa (SSA) where 80% of urban households use charcoal as their primary cooking fuel (Zulu & Richardson, 2013). Charcoal is dominated by two types of production systems (i) small‐scale producers including small‐scale agriculturalists who produce charcoal to supplement their income, often as a byproduct of land clearing for agricultural production, and by (ii) businesses in highly organized operations where transportation and sales chains are tightly controlled. Although improved charcoal cookstoves improve combustion efficiency and reduce the quantity of charcoal used per cooking event, no improved charcoal stove tested in a field setting has demonstrated significant and reliable exposure reductions sufficient to improve health against baseline technologies. In addition, reliance on charcoal for cooking has other negative externalities including contributing to deforestation and forest degradation in locations where production is intensive (Chidumayo & Gumbo, 2012).

Kerosene is also not included among our list of clean fuels because it produces harmful levels of HAP and safety risks for household users from handling and from accidental fires (Department of Health and Human Services, 2017). Kerosene combustion emits air pollutants, including PM_2.5_, nitrous oxides, sulphur oxides, and polycyclic aromatic hydrocarbons (PAHs). Combustion pollutants associated with kerosene use are linked to higher levels of tuberculosis, low birthweight and child pneumonia (Lam et al., 2012). While kerosene is sometimes mistakenly considered a clean fuel, and is still used extensively for cooking in India and urban areas of SSA for both cooking and lighting, WHO and the environmental health community strongly recommend against its use (WHO, 2014) and have clearly articulated that it is not a clean fuel.

Numerous countries have reduced or entirely removed kerosene subsidies to reduce the household use of kerosene. This has been motivated by the high cost of kerosene subsidies relative to other fuel options and because of the practice of using kerosene to adulterate non‐subsidized diesel and gasoline fuels. Indonesia aimed to phase out domestic use of kerosene completely by replacing it with LPG, beginning in 2007, reducing kerosene use by 92% over 10 years (Thoday et al., 2018). Kenya has programs that replace kerosene lanterns with solar lamps and simultaneously removed kerosene subsidies in order to promote LPG for household use (IMF, 2013). Ecuador reduced kerosene subsidies and transitioned from kerosene to LPG for cooking (from 20% of households use kerosene in the1970s to <1% of households at present) (Gould et al., 2018). In Bangladesh, the substantial roll‐out of solar home systems by the government agency IDCOL, induced a substantial decline in use of kerosene (Ishrat Malek et al., 2015).

Our analysis of clean fuel supply chains and enabling environments rests on a fundamental difference between solid fuels and clean fuels. Traditional solid fuels are often sourced locally and for free, or at low cost, from forests, woodlands and agricultural areas, providing many rural and some urban households with a readily available free fuel source (Angelsen et al. 2014). Household labor is the primary cost associated with traditional fuel collection. In urban settings and in some rural areas in LMICs (i.e., typically those characterized by scarcity of forest resources), there are markets for solid fuels. Clean household fuels are typically only free to consumers in cases where governments have committed to a 100% subsidy. Most clean fuels are imported (e.g., LPG), need to be produced locally in relatively nascent markets (e.g., pellets, biogas, ethanol), or require massive investment in infrastructure (e.g., electricity) (Table 1).

**Table 1:** Production and sourcing of clean household fuels

	LPG	Biogas	Alcohol fuels	Compressed biomass pellets	Grid electricity	Photovoltaic electricity
Production	By‐product of natural gas extraction and oil refining. Production starts at gas and oil wells where liquids are separated.	Animal and vegetable waste converted, typically in a household scale biodigester, to methane and piped directly into household appliance.	*Ethanol*: biomass fermentation using feedstocks rich in sugar or starch or cellulose; *Methanol*: gas synthesis from CO_2_, CO and H obtained from biomass or fossil fuels.	Wood, straw, sawdust or other wood‐ or plant‐based material, compressed using mechanical equipment.	Generated by electric power plants using natural gas, coal, solar, wind, hydro or other sources of energy.	Generated by photovoltaic cells.
Sourcing	Local when LPG produced domestically; most often imported through complex supply chains.	Highly localized.	Local when existing feedstock available, often produced from residuals; imported in some cases.	Local when existing feedstock available, often produced from residuals; imported in rare cases.	National scale infrastructure for grid connections; more localized sourcing for off‐grid unless distributed generation feasible.	Household level or scaled to municipal installations of smaller or larger sizes.

In sections 3‐5 we analyze the potential for implementation and scale‐up of clean fuels based on components (i)‐(iii) of the Logic Model, described above, with special emphasis on each component of dimension (ii). First we describe key characteristics of each clean fuel, including aspects of physical infrastructure for production, storage, distribution and practices (corresponding dimension (ii)); we then discuss the factors that influence the viability and sustainability of supply chains (Section 4) (also corresponding to specific aspects of dimension (ii)), and finally we review key elements of the enabling environment that influence scale‐up and user adoption of clean fuels, including fuel pricing policies, government policy and other aspects (Section 5) (combining dimensions (i) and (iii) together).

## 3 Description and Physical Infrastructure Requirements for Production, Storage and Distribution of clean Fuels

### 3.1 Liquefied Petroleum Gas (LPG)

LPG, is a naturally occurring and unavoidable by‐product of oil refining and natural gas extraction. LPG consists of a varying blend of propane and butane that can be stored in pressurized containers (steel or composite cylinders) (Bizzo et al., 2004). Because of its portability, LPG can be easily and safely made available and distributed in urban/peri‐urban settings and can become relatively accessible in rural areas, once supply chains are established.

LPG has been used for almost a hundred years as a cooking fuel (Poten & Partners, 2003) and continues to be popular in both developed and developing countries due to its high combustion efficiency (50%‐60%), cleanliness, and environmental performance (Bruce et al., 2017; Shen et al., 2018; Singh et al., 2017). Over 2.5 billion people in developing countries—43% of the global population—use LPG for some share of their cooking (IEA, 2017). In developed countries like Japan, LPG is the most widely used cooking fuel (WLPGA, 2017, 2018).

Global LPG production has been steadily increasing over the past decade with only 44% used in the residential and commercial sectors. A surplus of LPG exists and nearly 30% of global LPG production is used as a feedstock for plastics generation (WLPGA, 2018).

#### 3.1.1 Physical Infrastructure

The LPG supply chain starts with production at oil and gas wells. Unrefined oil and gas is shipped to refineries and gas processing plants (upstream transportation). It can then be stored or sold in its refrigerated or pressurized form. The LPG is then transported to downstream bulk storage terminals. It is further moved by road or rail (downstream transportation) to cylinder filling stations or 'bottling plants' for bottling and storage. This is the stage where LPG is transferred to cylinders and distributed to the residential or commercial sectors. LPG for household fuel use is sold in cylinders ranging in size from 3 to 15 kg. Commercial‐use cylinders (e.g. used in restaurants) range from 25 to 50 kg. Cylinders are typically available in different sizes to meet local market needs, with smaller sizes (3–6 kg) being more affordable for low‐income households but more easily damaged (WLPGA, 2018).

#### 3.1.2 Distribution

LPG cylinders are delivered to the end‐users through a complex distribution system involving wholesalers, dealers, agents and retailers. Distribution generally occurs through cylinder delivery trucks that move the LPG from bottling plants to local distribution/retail points near the end users.

Depending on the country and extent of the LPG market, companies may offer LPG delivery and after sales services with 24‐hour emergency hotlines (e.g., India, Brazil). In India, the doorstep delivery model in urban centers has positively influenced LPG usage (Jain et al., 2018). However, this service is not yet extensively offered to rural areas, where users still need to invest in time and travel to reach the closest retail point to exchange their empty cylinder for a filled one.

A number of recent national programs have seen LPG distribution reaching millions of households in just a few years, as occurred in Indonesia (2007‐2010) (Thoday et al., 2018), and in India (2015‐2019) (Goldemberg et al., 2018). Other countries have historically reached scale over longer periods of time (e.g., Morocco and most Latin‐American countries) (Troncoso & Soares da Silva, 2017; WLPGA, 2013).

#### 3.1.3 Rules, Safety Regulations, and Practices

In order to ensure a high and consistent level of safety for supply chain agents and end‐users, strict safety rules need to be applied. In most countries, LPG storage, transportation and handling are strictly regulated at the national level by one or more regulatory entities (GLPGP, 2015).

Cylinder ownership influences the safety of the LPG market and its potential for scale. The 'branded cylinder recirculation model' (BCRM), where cylinders are owned, inspected and maintained by the LPG marketers, is the first key step in developing a sustainable and safe domestic market. Under this model users pay a deposit to obtain a cylinder and to continue to exchange their empty cylinder for a filled one of the same brand. LPG marketers are responsible for maintaining/replacing their own branded cylinders to ensure optimal performance over time, as well as for cylinder distribution and last‐mile reach. The BCRM is the market model in the majority of countries around the world. In contrast, countries operating the 'customer‐controlled cylinder model' (e.g., Nigeria, Guatemala) frequently experience a decline in safety of existing cylinders because no LPG marketer is responsible for cylinder inspection and maintenance, and illegal and unsafe filling activities can proliferate (WLPGA, 2013).

Under the BCRM, only the LPG marketer can authorize the transport of LPG in bulk or in cylinders, and distributors, transporters and selling agents must be licensed. To ensure end‐user safety, cylinders and valves need to be inspected regularly and strict rules and penalties to avoide illegal filling practices need to be in place (WLPGA, 2013).

LPG is safe to use but requires careful handling. Warning of leaks is made possible by the addition of an odorant (Bizzo et al., 2004). Leaks can occur from the cylinder body, valve or rubber hose that connects the cylinder to the stove. Because LPG is heavier than air, it will descend to the ground where it may be ignited by an electric spark, discharge or a cigarette and result in a fire. Correct placement of the cylinder, in an upright position, and stove, above the cylinder and never on the floor, is important for the prevention of accidents (GLPGP, 2017). Protocols in road transport must be followed to avoid accidents, which could lead to fires and explosions.

### 3.2 Biogas

Biogas is a mixture of gases (primarily methane) derived from anaerobic digestion or fermentation of manure or organic waste. Biogas from household‐scale digesters is a feasible technology in LMICs with significant potential for expansion in rural areas with ready access to animal and agricultural waste, as well as water, to feed digesters. The technology has spread mainly in Asia (Chen & Liu, 2017; IRENA, 2017; Mittal et al., 2018) and only modestly in Africa (Amigun et al., 2012; Clemens et al., 2018; Roopnarain & Adeleke, 2017) and Latin America (Fundación Ecología y Desarrollo, 2016).

Sustained biogas use is dependent on both continuous supply of biodigester feedstock and consistent daily operation (Puzzolo et al., 2016). To produce cooking gas for a household of four people, a daily amount of 20 kg of manure is sufficient. Typically, two cows or seven pigs produce sufficient manure (Ghimire, 2013; IRENA, 2016; Lisowyj & Wright, 2018). Combining animal manure and food residue substrates leads to higher energy potential than animal waste alone. However, improperly mixed waste creates a risk of inhibition of the biogas generation processes (Lisowyj & Wright, 2018), and sometimes suppliers recommend the use of manure only.

#### 3.2.1 Physical Infrastructure

Digesters are built or installed at the household level as either a fixed dome, floating drum, or in a prefabricated form, and operations include frequent (daily) supply of new feedstock as well as occasional maintenance and repair. Fixed dome digesters have been promoted by donor agencies (e.g., the German GTZ, the Netherlands Development Organization (SNV)) as being robust and having a lifetime of 20 years or longer if properly maintained. Prefabricated digesters are increasingly popular in China (Chen & Liu, 2017) and Mexico (Fundación Ecología y Desarrollo, 2016). In both cases the materials for the digester are readily available. A small land area and access to water are also required. Biogas appliances and connectors (stoves, piping, valves) are produced in countries with larger markets (e.g., China, Vietnam) and imported into areas with fewer customers (e.g., Africa). Some households attach modified LPG cookstoves to a biodigester, but these often result in lower efficiency.

#### 3.2.2 Rules, Safety Regulations, and Practices

Biogas markets are emerging, but are rarely well established. These emerging markets have been supported by donor programs including those from the World Bank and SNV. SNV has supported numerous programs through a sector development model, initially in Nepal and later replicated in Asia and Africa (Ghimire, 2013). Biogas introduction requires that national governments (e.g., the Ministry of Energy) provide policy, legal, and institutional frameworks, while the private sector is incentivized to install biodigesters by skilled technicians. As biodigesters have multiple uses (biogas, bioslurry, waste treatment), the institutions supporting biogas uptake can vary and may be several (e.g., Ministry of Energy, Ministry of Agriculture and Livestock, etc.), which may complicate the introduction of enabling policies to support dissemination at scale. Quality control and standards development are critical to trigger an evolving market. Research and development, training and demand creation are needed in a starting phase of sector development (Ghimire, 2013). Biogas and biodigesters are generally considered safe to use and operate.

Evaluations of household biodigester programs show high levels of client satisfaction and functionality in several countries (Bajgain et al., 2005; Bedi et al., 2015; Bedi et al., 2017). In Cambodia, a survey of 165 biodigester users, 96% were satisfied with their biodigester and 100% use the biogas for cooking (Hyman & Bailis, 2018), similar to other biogas surveys. In East Africa household level biodigester usage rates range from 67% to 77% of those installed (Clemens et al., 2018). Users report a variety of reasons for non‐functioning of biodigesters, including improper provision of feedstock (perhaps related to a lack of training). This has led to fuel stacking with traditional solid fuels (Clemens et al., 2018). Rural modernization is affecting smallholder livestock ownership in countries with draft animals, and this may eventually undermine demand for biodigesters in some settings (Hyman & Bailis, 2018).

### 3.3 Alcohol Fuels

These comprise ethanol and methanol (see section 3.3.1). Ethanol is obtained from diverse sources but is mostly produced from biomass feedstocks rich in sugar or starch or from cellulosic feedstocks. Starch and cellulose are broken down by enzymes and transformed by yeasts into ethanol. Cellulose degradation requires an extra step, acid hydrolysis, to prepare it for treatment with enzymes, prior to distillation. Ethanol may also be synthesized from natural gas, coal or ethylene, a by‐product of petroleum. Other feedstocks, such as municipal solid waste, are sources of carbon for synthetic ethanol (Enerkem, n.d.). New technologies are emerging that produce ethanol through alternative pathways. A hybrid process uses anaerobic bacteria to ferment ethanol from CO in synthesis gas (Handler et al., 2016).

Ethanol can be produced in both large and small plants using conventional fermentation technology. Cellulosic and synthetic ethanol require large plants and economies of scale. More than 100 billion liters of ethanol are produced annually around the world. The International Energy Agency projects that this will exceed 145 billion liters by 2024 (REN21, 2018).

Ethanol requires specialized stoves. A high‐performing liquid ethanol stove was developed in Europe for the leisure market in 1979 and was introduced to Africa in 2001 (Stokes & Ebbeson, 2005). Ethanol fuel attracted the interest of the Ethiopian government as a way to develop a market for ethanol produced in state‐owned sugar factories. Ethiopia was one of the first countries in Africa to adopt a biofuels strategy, identifying ethanol as cooking fuel and the stove market as a strategy for developing its ethanol sector (Benka‐Coker et al., 2018).

#### 3.3.1 Physical Infrastructure

Ethanol in LMICs is most often produced from molasses, a by‐product or residue of sugar production. Many sugar factories in LMICs do not have distilleries. While some molasses can be used in animal feed, most is considered waste material and is disposed of through land application. When a distillery is co‐located with a sugar factory, it receives steam and power from the factory in addition to molasses to make ethanol.

In contrast with molasses‐based distillation, stand‐alone distilleries may also operate that produce ethanol from a wide variety of feedstocks, including sugarcane, cassava, tropical sugar beet and grain sorghum. Nigeria, the world's leader in cassava production, produces ethanol from cassava, as do Indonesia, Vietnam and Thailand. Cassava to ethanol projects are underway or have recently opened in Sierra Leone, Ghana, Uganda, Zambia, Malawi and Zimbabwe (Oketch, 2016; Sunbird Bioenergy, 2019).

#### 3.3.2 Distribution

Ethanol is a liquid fuel handled and moved in a manner similar to kerosene. Kerosene tanks for storage or transport can be repurposed to ethanol (STI‐SPFA, 2012). Finished ethanol for the household market is moved from the distillery in tanker trucks of 30‐50,000 liters. Prior to distribution, denaturants are added (e.g., denatonium benzoate and color).

A filling plant may be located at the distribution depot. Ethanol fuel may be distributed in polyethylene terephthalate (PET) or high‐density polyethylene (HDPE) multi‐use containers, sold in various sizes. There is currently experimentation with ethanol fuel pumps and dispensers in Kenya that allow a consumer to pay for a specific amount of ethanol using mobile money (Dalberg, 2018).

Though most ethanol is produced in country, ethanol can be imported to markets by overland trucks or tanker ships in ISO‐tanks or bulk loads.

#### 3.3.3 Rules, Safety Regulations, and Practices

Alcohol fuels are relatively new to most LMICs. As a result, laws and regulations governing their use are less well developed than for petroleum fuels. Historically in LMICs, ethanol has been produced and taxed for the beverage market. These taxes are set much higher than taxes on fuels. If ethanol is to be used for fuel, a clear regulatory distinction at the point of manufacture must be made between alcohol for beverage uses and alcohol for fuel. This begins with proper denaturing, as set forth in regulation.

Many countries have yet to create a regulatory definition for ethanol fuel. Kenya represents a coherent model to be replicated elsewhere. Kenya has developed a regulatory framework for ethanol cooking fuel (Project Gaia, 2016). An ethanol stove business in Kenya must register in the Office of the Attorney General and obtain a certification from the Kenya Revenue Authority, which manages tax reporting. A license must be obtained from the National Environmental Management Authority (NEMA). The Kenya Bureau of Standards (KEBS) evaluates fuel quality and safety and approves standards for packaging and distribution. Finally, the Kenyan Industrial Research and Development Institute (KIRDI) evaluates the stove and provides documentation to the KEBS and the public. For a fuel distributor to offtake from any of the Kenyan distilleries, it must show its required permits. Local jurisdictional rules also apply.

Ethanol is volatile and must be handled throughout its supply chain to avoid release of vapor in explosive concentrations, described by Lower (LEL) and Upper (UEL) Explosive Limits. The flammable range for ethanol vapor is between 3.3% and 19% in air. The LEL for butane (1.8%) and propane (2.1%) are lower than ethanol, but the flammability range for ethanol is wider (Engineering Toolbox (n.d.)).

International standards must be established for ethanol stove fuel. When ethanol is produced through distillation, it contains small amounts of higher alcohols that generate soot when burned. These impurities can be removed during distillation. ASTM International has established a standard for cookstove fuel, ASTM 3050‐16, which addresses both the ethanol and the denaturant to be added (ASTM International, 2016). This standard was designed with reference to blend‐stock ethanol produced in fuel distilleries in the U.S. A cleaner standard remains to be developed.

Alcohol stoves also require standards. The development of ISO standards for stoves is an important step in this direction. The alcohol stove introduced to Africa in 2001 was designed to hold ethanol in a canister with adsorption onto the surface of a mineral fiber to prevent leakage. Alcohol evaporates from the canister into a combustion chimney where it mixes with air and is burned. The flame is controlled by a regulator that slides across the evaporative surface of the canister (Jetter et al., 2015). When the adsorbent fuel canister technology was invented, it quickly replaced other alcohol stoves that required pressure or the use of gel fuel.

#### 3.3.4 Methanol

Methanol, once known as wood alcohol, is today largely produced from natural gas. It can be produced from any carbon source. In Iceland, CO_2_ from a geothermal power plant is reacted with hydrogen to produce methanol (Carbon Recycling International, n.d.). Direct Air Capture of CO_2_ is an emerging technology for methanol production (Keith et al., 2018). Global methanol production is about 130 billion liters per year, with an annual growth rate of 5% (Methanex, 2018).

China is the world's largest methanol producer. Replacing coal with methanol has become a strategy of several cities in northern China to reduce air pollution. Gansu Province and Tianjin City have enacted policies to encourage methanol for cooking. The development of commercial and institutional methanol stoves has led to a surge in methanol demand, with consumption at 3 million metric tons and expected to reach 5 million tons by 2020 (Zhao, 2018).

In India, the government's national planning committee is encouraging development of household cookstoves using methanol. An Indian producer, Assam Petrochemicals, has launched a methanol cookstove project in Dibrugarh District, Assam State (Chakraborty, 2018; Saraswat & Bansal, 2017). In contrast to ethanol, methanol is not safe to handle and must be properly contained to minimize contact. The Assam project uses the adsorptive fuel canister for this purpose. Fuel is distributed in the canister, which is sealed. The seal is removed when the canister is placed in the stove.

The utility of methanol as cooking fuel depends upon it being packaged safely for consumers to handle (methanol is toxic to human by ingestion, inhalation or absorption through the skin). In much the same way that gasoline or kerosene must be managed to avoid skin contact or inhalation of vapors, methanol must also be managed to avoid skin contact and inhalation. The time‐weighted average (TWA) and short‐term exposure limits (STEL) values for methanol are similar to those of gasoline and kerosene, but much lower (i.e., more restrictive) than for ethanol (Methanol Institute, 2017).

### 3.4 Pellets (When Used With Low Emission Stoves Tested Under Field Conditions)

Biomass compression, when carried out to produce a consistent product, can reproducibly form a solid biofuel that has low moisture content and high energy density. Compressed biomass fuels are capable of clean combustion (i.e., low pollutant emissions) when manufactured to high standards and used as intended in stoves designed to meet Tier 4 or better performance targets for emissions and efficiency (Champion & Grieshop, 2019). Depending on the size and shape of compressed biomass, it may be called pellets or briquettes, with the latter being the larger of the two. For the purposes of this analysis, the term “pellet” is used to refer to pellets and briquettes.

Pellets may be formed from a wide range of unprocessed, untreated biomass feedstocks, including wood and branches, sawdust, straw, and crop residues. Typically, the raw materials are byproducts or waste products of agricultural or forestry‐related activities.

#### 3.4.1 Physical Infrastructure

Biomass pellet production is a semi‐mechanized process. The core production step involves compression and extrusion of the pellet. To support this step, a production system typically includes machines to reduce the size of the raw materials and convey materials within the processing facility, furnaces and facilities for drying the raw material prior to processing, devices for cooling and adjusting the moisture content of processed raw materials, and machines for capturing and packaging the final product. Pellets are best stored under environmentally‐controlled, cool, and dry conditions to prevent the growth of fungus or other contaminants.

Production of pellets for residential cooking can be either decentralized or centralized. Having decentralized production facilities near communities relies on the availability of pelletizing infrastructure in numerous locations. Distributed production minimizes both feedstock and pellet transportation and distribution costs (see below) but requires capital investment and operation and maintenance of multiple facilities across a wider geography. Centralized facilities can take advantage of economies of scale, but at the expense of requiring a pellet distribution infrastructure. There is an inherent tension between scale‐up and the required capital equipment needed to establish a pellet supply chain (observed in both Rwanda and Zambia where single pellet factories are supplying limited markets in the entire country) (Jagger & Das, 2018). Conversely, production of pellets in China and in other developed countries is often centralized, in part due to established feedstock supply chains, relatively low‐cost transportation systems, and economies of scale (Carter et al., 2018)

#### 3.4.2 Distribution

Transportation systems for pellet production are needed both to bring raw materials to processing facilities and to distribute the final product to end‐users. Sourcing of feedstock from a small number of large suppliers is much more efficient than sourcing of feedstock from a large number of small suppliers. In Rwanda, the only firm producing pellets for use in residential cooking started out with a feedstock supply model that involved rural households sourcing and delivering feedstocks to local depots. This rural feedstock was then transported onward to the pellet factory. As the company has continued to scale‐up, they have had to source feedstock from larger suppliers, making the transport of feedstock considerably less complex (Jagger & Das, 2018). Similarly, in Zambia, where there is one firm supplying the local market, sawmills and medium‐scale agricultural processing facilities play a major role in feedstock provision. A similar model was used for a pellet‐based stove company in India, where feedstock came in the form of waste from an agricultural processing facility (Thurber et al., 2014). Minimizing the transportation cost associated with sourcing feedstock is an important aspect of the business model of these firms.

Distribution of pellets, like any other processed fuel, requires a reliable distribution system and is critical for the widespread use of the fuel. Biomass processing can be established to deliver pellets in small batches to homes. For example, in both Rwanda and Zambia the private sector firms that promote pellets deliver them directly to households that subscribe to pellet packages, in addition to having point of sale retail outlets at petrol stations, grocery stores and at their own retail outlets. In India, Oorja established a dealer network (sometimes in partnership with others) that was responsible for stove sales and aftermarket support and sales of pellets (obtained by higher level distributors) (Thurber et al., 2014). Other distribution models require that households travel to the biomass processing facility using their own means of transport to purchase pellets. Distribution systems for pellets in rural areas are a challenge, particularly in cases where the density of users is relatively low (Shan et al., 2016). In addition to often limited road infrastructure, seasonal fluctuations in the time and effort required to deliver pellets, and availability of reliable vehicle fleets, simple economies of scale make distribution of pellets to rural areas often untenable.

#### 3.4.3 Rules, Safety Regulations, and Practices

Industry standards and private sector governance for pellet production operations in LMICs are limited. In China, however, there are notably multiple professional societies and government organizations associated with the manufacture and dissemination of household stoves for cooking and heating, some of which focus more heavily on pellet manufacturing. For example, one professional society of pellet manufacturers engages in collective efforts to solicit increased government involvement and action in the pellet market through improved access to higher quality machinery, increased subsidies to households to buy pelletized fuels and accompanying stoves designed for burning pellets, and incentivized collection of crop residues by banning crop field burning (Carter et al., 2018).

Some efforts within pellet manufacturing markets have focused on setting limits for the proportion of non‐biomass materials (e.g., plastic trash) that can contribute to the overall pellet content. In one extreme example from a southern province in China (with low heating demand), biomass pellet production was banned altogether because of the perceived inability to maintain sufficient quality control, leading to production of non‐biomass pellets capable of emitting high amounts of harmful and carcinogenic air pollutants (Carter et al., 2018). Some countries have introduced sustainability certification schemes for pellet production for use in higher efficiency stoves (e.g., Japan, Korea), but this does not seem to have expanded to LMICs (Thrän et al., 2017).

African firms face an unclear regulatory environment with few regulations or regulations designed for other aspects of energy or manufacturing sectors. African national standards organizations (e.g., Rwanda Bureau of Standards, Malawi Bureau of Standards) are beginning to use ISO guidance in the regulation of improved cookstoves. In countries where national standards for cookstoves are evolving, to the extent that they involve pellet stoves such as Tier 4 rated forced‐draft micro‐gasification stoves, regulations and standards for the pellet fuel should not be far behind. These rules and standards that cover both the stove and the fuel are critically important given the differences in performance that have been seen between laboratory studies and field measurements (Champion & Grieshop, 2019; Wathore et al., 2017). If variability in stove performance in the field can be reduced through standards development and enforcement, the air pollutant reductions anticipated based on of low emission laboratory performance is more assured.

Rules and standards regarding pellets should also address concerns that can arise even prior to pellet combustion. Pellet safety concerns range from high occupational exposure to dust and particulate matter during production to fire and possible fungal growth if stored in sub‐optimal conditions. Existing safety standards have been developed in North American and Western and Northern European countries to address these concerns, as well as fuel and stove performance, and could be shared to improve pellet safety for both production and use (WHO, 2015).

### 3.5 Electricity (Grid and Photovoltaic)

Electrification is a topic that extends well beyond the discussion of clean household fuels, but aspects of electrification are critical in determining the relevance of electricity for household cooking. At point of use, electricity is a clean fuel for household cooking.

Grid electricity is feasible when there is both proximity to the grid as well as grid capacity and transmission capacity. In some urban settings, for example Lusaka, Zambia, grid electricity is a relatively common source of cooking energy for households wealthy enough to have grid connections. In South Africa, as the majority of households have transitioned to cooking with electricity, the erratic nature of the electric supply, exacerbated by the growing demand, overwhelmed the grid and led to increasing reliance on other fuels (Ateba et al., 2018; Israel‐Akinbo et al., 2018). In rural areas of SSA which have not yet achieved 20% electrification and where transmission distances are large, grid‐based electricity may not be a viable cooking solution.

Minigrids based on photovoltaic systems offer a potential alternative to large scale grids in these areas where grid electricity is not feasible. Also, in some regions, a strategic mix of on‐grid and off‐grid solutions, with a substantial contribution from photovoltaic‐fueled micro‐ and mini‐grids, may be the best electric solution for cooking.

The amount of electricity required for cooking is high. The FAO estimates that electricity as a cooking fuel requires 3232 kWh per year per household (Kammila et al., 2014), equivalent to over 8 kWh per day. These values are likely on the higher side of cooking requirements in LMICs. By increasing end‐use efficiency of the cooking process, for example by using rice pressure cookers or pot warmers, the total energy requirement may be reduced. Household cooking practices could impact energy requirements dramatically (Kar et al., 2018).

#### 3.5.1 Physical Infrastructure

Photovoltaic systems can be highly customized and can be sized for individual households or communities. Photovoltaic systems can be designed to match user needs by varying delivery capacity, storage size, community distribution systems, and targeted cooking appliances, but must meet the needs of the user (Brown et al., 2017). Furthermore, communities that collectively build photovoltaic systems can use a simple distribution switch to share, and separately bill for, known quantities of electricity across households. Households or communities can install a first‐generation system that shifts some cooking tasks to electricity, even in the absence of any battery storage in the mini‐grid, by time‐shifting cooking tasks to take place during the afternoon.

Batteries for energy storage, purchased at current prices, typically double the installation cost of the system and increase dependence on operation and maintenance. Innovations abound in energy storage and may lead to affordable and reliable storage through more advanced self‐balancing battery systems, higher tolerance to rapid cycling, and more robust responses to energy drawdown.

Cost estimates for photovoltaic cooking vary substantially based on estimated energy requirements, generation efficiency, distribution distance, required energy storage for late day cooking, and expected economies of scale for the system installations. In order to ensure 24‐hour electricity access from photovoltaics, storage systems such as batteries or innovative energy storage systems are necessary and add to the cost (Brown et al., 2017). If access to alternating current is important, then inverters and the associated energy losses add to the cost. Efficiencies can be achieved by using cooking appliances that complement the energy delivery system. For example, rice cookers, pot warmers, and slow cookers are used to improve efficiency when electricity supply is limited. New and efficient electric cooking technologies suggest that when electricity is planned and coordinated effectively, costs can be reduced to as low as $0.21/kWh, and the average cost of cooking a meal may be reduced to $0.33/meal (IEA, 2018). These costs are competitive with other clean fuels such as LPG and ethanol.

#### 3.5.2 Practices

To create a functional market for investment in lower‐cost mini‐grids, rural electrification agencies must be strengthened (Trotter, 2016). Policies must be developed to support the private sector electricity market participants, especially since they may be better positioned to improve power availability than central planners (Kessides et al., 2006).

Perhaps the key to developing mini‐grid electric systems for cooking is the recognition that, while generating electricity is simple and well understood, distribution algorithms and cooking appliances require innovation. Induction stoves have typically required 1 kW of AC electricity, but new designs are able to use 24V low voltage DC by using a resonant converter to generate the oscillation for the induction stove and still achieve efficiencies of 90%, without requiring an inverter to create AC electricity (Weber, 2014). Although these DC‐powered induction stoves could be connected directly to DC solar power, they still require substantial electricity (e.g., hundreds of Watts), and this level of solar power generation requires up‐front capital investment that will likely also require financing solutions to distribute that investment over time. These stoves would also be useful with battery backup power in grid‐based systems that experience brownouts. Using electricity at the point of generation through mini‐grids will require innovation beyond that of the grid electric systems.

## 4 Contextual Factors of a Sustainable Fuel Supply

In developing countries, clean fuels, including electricity, may be newer, less familiar choices for cooking than traditional fuels; they may have underdeveloped production and supply systems and fragile, nascent markets. These cooking systems are new and untested for the consumer, and may be perceived as not meeting all of the culture‐specific cooking needs. This section addresses risks to supply and demand that may be critical factors for moving from an emerging energy service to an established one, with reliable supply and demand. Reliability of supply will affect demand. Key factors affecting fuel supply are presented in Table 2.

**Table 2:** Supply chain factors for clean household fuels

Factors	Description
Feedstock supply availability	Typically, production costs are highly dependent upon feedstock availability (procurement and transportation, as applicable) as well as feedstock conversion to fuel. Fuel production costs are influenced by installed technology, automatization and economy of scale.
Feedstock price variability	Lack or scarcity of feedstock impacts the full supply chain and ultimately the ability of end users to rely on such fuel for their daily energy needs. When local feedstock and production become constrained, imports may be required to meet demand. To reduce risks due to importation, import agreements and mechanisms to mitigate fuel prices are necessary.
Safety	To ensure a consistent, high level of safety for operators and end‐users, governments, local authorities and companies must define and enforce national standards, laws and regulations (including licensing as applicable), as well as inspections and operational practices for equipment.
Accessibility and last‐mile distribution	Reliable and extensive distribution infrastructure (e.g., power grids or roads) is a prerequisite for creating access to most clean fuels. Decentralized solutions (biogas, PV, small pellet plants or distilleries) are better suited for last‐mile distribution in rural settings but require access to operational and maintenance services. In addition, households need access to points of sale where the fuels can be purchased. Long distances to points of sale, especially in rural areas, can limit adoption and widespread use. Home delivery for fuels like LPG and pellets is practiced in high‐population‐density areas.
Scale‐up potential	Some fuels are directly linked to national decision‐making and infrastructure development (e.g., LPG, grid electricity, ethanol production) while others may rely on local decision‐making and smaller scale infrastructure and operations (e.g., pellets, biogas, ethanol). Potential to reach scale depends on multiple factors, including feedstock availability, economically viable and reliable operational production and distribution, as well as appropriate policy (see Section 5).
Convertible currency or foreign exchange	Where fuel, appliances and accessories must be imported, a stable, convertible currency, or government commitment to providing foreign exchange, is a factor in ensuring continuous supply.

Each fuel has its constraints and opportunities The uninterrupted supply of fuels and the appliances and accessories to use clean fuels are essential to ensure adoption and sustained use at the household level. For example, pellets and ethanol are based on agricultural and/or forest products that may vary with season and weather conditions, crop productivity, and even road access and require a supply of labor and machinery to ensure consistent supply. Pellets and ethanol production are embedded in the agricultural economy and can affect the market for agricultural products and residues. Similarly, ethanol production may affect molasses pricing for supplementing animal feed or may impact the cost and availability of molasses for breweries. It will add new income to sugar operations and may increase sugar production. Challenges for pellet production include: (i) sourcing of feedstock, (ii) acquisition and deployment of pelletizing equipment, including operations by personnel often limited in technical experience; (iii) equipment assembly and maintenance such as replacing pellet dies; and (iv) storage and drying of pellets, particularly in humid climates.

For nascent or early‐stage LPG markets, fuel shortages are often common and are key market disruptors. Supply chain constraints are often the result of a lack of adequate policies, regulations and/or regulatory enforcement to stimulate and sustain marked development, including investment in cylinders (Van Leeuwen et al., 2017). If LPG and cylinders are imported, commodity pricing fluctuations, local currency inflation and shortages in foreign currency exchange can influence fuel supply.

The physical and chemical properties of fuel, their energy density, their ability to be transported and stored, all affect the cost of delivery. Distribution is key for all fuels that need to be physically transported to reach the end‐users. Distribution networks are typically most efficient in urban centers where consumers are more densely clustered; as a result, rural areas are often underserved. Domestic biogas, on the contrary, is better suited in a rural environment where biogas installations are mainly family‐sized plants and generate gas for domestic use (Cheng et al., 2014). However, larger‐scale commercial biogas systems that treat organic waste from municipalities, large livestock farms, large plantations/crop farms and then distribute to households through piping are also a technological option to be further expanded (Kemausuor et al., 2018).

Electricity has a specific set of distribution considerations including whether the electricity is generated in solar mini‐grids or from large scale power plants with extensive grid distribution systems. Affordability and consistent supply are also key considerations. Thus, subsidies, financing programs, consistent supply chains and financial structures that impact affordability and availability remain critical. Furthermore, since the installation of electric generation is capital‐intensive and requires distribution and maintenance, a centralized approach where individual households are not responsible for the utility is imperative. Thus, key enabling factors include strengthening rural electrification agencies and developing a functional market for minigrids (Trotter, 2016).

## 5 Contextual Factors of the Enabling Environment

Creating a strong enabling environment for the transition to clean household fuels requires a combination of national planning, policy reform, financial agreements, regulatory decision making, and targeted investments across the fuel supply chain. Strong government support is needed to help set the conditions for adequate and stable market rules and the ability to enforce regulatory measures. Pricing regimes, taxes, subsidies and market adjustments must be tailored to enable lower income households to adopt and use the fuels consistently. Financing for subsidies can sometimes be fully or partially offset by development agencies, governments, special finance vehicles, and results‐based financing, including carbon credits. Subsidies can be justified (and paid back over the long term) by public benefits, for example reduction in urban air pollution or improvements in health. The enabling environment should eventually include standards development as well as processes and protocols to ensure quality control.

Some contextual factors are inherent in the supply chain and are included in the individual fuel descriptions above (e.g., specific safety regulations for fuel supply and distribution elements). Additional contextual factors, including the political and economic contexts of a country that influence the enabling environment for the transition to clean household fuels, are presented in Table 3. These contextual factors broadly include government policies and regulations for the energy/cooking sector, financing requirements, as well as fuel pricing policies including taxation and subsidies, which ultimately affect end‐user costs.

**Table 3:** Contextual factors of the enabling environment

Factors	Description
Policy	The development of a comprehensive national energy policy framework, including household energy needs, requires cooperation across government ministries (e.g. energy, finance, health, environment, agriculture, petroleum, etc.). Policies need to address rules, standards, and best practices to support long‐term fuel market expansion. Policies supporting a ‘fuel of choice for the household sector' can accelerate market expansion and meet demand.
Regulation, standards, and certification	Regulation, certification, standards, and enforcement of rules are needed to promote sustainable supply and to reduce safety risks in handling/distribution and use. Regulation needs to be customer‐oriented, but still stimulate competition, and appropriate for the range of technologies, fuels and business models that can meet customer needs. At the same time, certain fuels need strong law enforcement to eliminate illegal practices (e.g., LPG, ethanol, electricity).
Infrastructure financing	Financing mechanisms are required to support physical infrastructure development and distribution. Consumer loans may also be helpful. Requirements for capital financing include: (i) lending institutions with capacity to provide mid to longer term finance for the construction of production facilities (e.g., terminals, distilleries, pelletizing equipment, biodigesters, grid connections etc.); (ii) multilateral banks prioritizing fuel projects through targeted lending and guarantees; (iii) creditworthy businesses with convincing business cases and written business plans to be eligible. In Africa, commercial banks and microfinance finance institutions (MFI) may have more reliable income from larger clients and may be reluctant to lend to consumers or households.
Capital cost customer financing	The stoves/equipment that use clean fuels also impose up‐front costs to the consumer that should be anticipated in comprehensive pricing supports. Low income households may need supportive financing to afford upfront and ongoing expenses. Examples of customer financing include: free test periods, warranties, leasing/credit offers, loyalty programs and more innovative models (e.g., Pay‐as‐You‐go).
Fuel pricing policies (including taxation and subsidies)	The purchase of any fuel, with the exception of domestic biogas, imposes a financial cost that can be significant when compared to household disposable income. The costs are highly dependent upon the national sourcing strategy and available natural resources. Transportation and distribution costs add to the final price and can be higher for rural users. Price variability and market development can be mediated by changing taxes, introducing price‐regulation mechanisms or subsidies. Market balancing may be necessary (e.g., imposing or enforcing taxes and regulations on fuelwood and charcoal). Competition for fuels in other markets such as transportation or industry can also influence fuel price.
Marketing strategies	Effective marketing provided by companies/fuel providers/the Government can contribute to increae customer awarnesses and overall fuel uptake. Promotion and education campaigns conducted in local languages and dialects can reach a greater number of households and a combination of televesion/radio adverts as well as pulbicity material has succesfully been employed in national‐scale programs (e.g. India, Indonesia).
Customer support	Customer support systems for operations and maintenance are essential for continued and sustained use of clean fuel. Decentralized solutions such as biogas and PV or small‐scale pellets or distilleries may be better suited for last‐mile distribution in rural markets, but require access to maintenance (e.g., biogas, ethanol, pellets) and storage services (e.g., PV electricity, ethanol, pellets). Equipment warranties and guarantees improve continued technology use. Industries/companies with ‘customer‐oriented focus' tend to be more successful. Overall quality control and standards are important to create consumer trust in the market.
End‐user awareness and behavior change	Socio‐cultural behavior and awareness of clean fuel options (including how to access clean fuels) are necessary critical elements for clean fuel take up. Furthermore, gender and the effects of gender on household decision‐making should also be considered in program and policy planning.

### 5.1 Fuel Economics

The economics of fuel in the supply chain is a key factor in scaleup, affecting the price of the fuel to the consumer and its competitiveness (or value for money) against other fuels.

The relative cost of a fuel is dependent on factors operating at three levels in the supply chain:

1. sourcing and/or production costs are based on the sourcing, production and processing, and the cost to get the fuel into the supply chain, including any import duties and taxes. Some fuels are also subject to market risk factors (e.g., prices at import being tied to international oil prices or fluctuations in biomass feedstock prices due to supply/demand forces).
2. distribution costs include both infrastructure costs and programmatic costs. Infrastructure costs include storage facilities and distribution (trucks, tankers, dealers, etc.). Programmatic costs include marketing the fuel to stimulate demand or to compete against other firms offering similar fuels. Marketing costs may be partially borne by the public sector (e.g., government or public interest marketing around health benefits of cleaner fuels) or by implementers (public or private). Additionally, training costs may be required to ensure proper use and handling of fuels and appliances.
3. consumption costs, comprising both techno‐economic factors of the fuel and behavioral factors influencing the perception of costs. Techno‐economic factors are the final price of the fuel and its appliance, additional costs (e.g., Pay‐as‐you‐go LPG requires a meter that may not have existed previously) and last‐mile costs (time and effort to reach the individual kitchen). These costs will be compared by the consumer to a baseline, or to what she or he has been accustomed.

Households will also be influenced by the variability in the fuel price and by a range of other behavioral factors (e.g., how households value the performance of the fuel and its stove compared to other alternatives). Supply of gathered fuelwood or preference for traditional cooking fuels and practices will influence the perceived value‐for‐money of any clean cooking fuel. Previous studies of determinants that affect household decisions for clean cooking choices identify affordability and consistency and reliability of supply as the most critical (Dalaba et al., 2018).

### 5.2 The Role of Policy in Fuel Costs and Prices: Financial Incentives, Disincentives and Market Conditions

The economics of fuels can be greatly influenced by policy and result in different consumer prices and ultimately different value‐for‐money calculations by consumers. For example, a recent study for SSA shows that for most clean fuels, the annual fuel cost for cooking far outweighs the annual capital cost of cooking equipment (PBL, 2018). Policy tools that affect fuel costs and prices can be categorized as: (i) provision of direct incentives (e.g., subsidies, deferred payments, montly installments, fuel vouchers), (ii) reduction in direct disincentives (e.g., eliminating taxes or costly regulatory barriers), (iii) reduction of direct incentives to existing polluting fuels (e.g., subsidies on kerosene), and (iv) alteration of market conditions (such as improving competition). Each is described below with potential issues that can result in negative outcomes.

It is important to note that the application of these policy tools has not been consistent across fuels, technologies, and geopolitical settings. A fuel or technology might be highly subsidized in one country while not given any preferential treatment in another country. Policy recommendations for specific household energy pathways must take into account the particular country context and the existing policy landscape.

#### 5.2.1 Provision of Direct Incentives

Direct incentives can be applied to either reduce initial equipment costs or to reduce ongoing fuel costs. These can include subsidies and tax abatements (uniform or targeted to certain household segments), equipment rentals (where fuel and equipment are bundled in one payment), and a variant on this, where equipment is paid off in fuel sales (causing the cost of the equipment to disappear), and other incentives, such as loans, microfinance (Zerriffi, 2011). Promotion of targeted subsidies to the lowest income households has been found to be a more effective use of public money than uniform incentives across the population. For example, in India, the government has reformed its subsidy program for LPG to: eliminate fraudulent connections and reduce diversion (subsidies now are deposited into beneficiary accounts through the Direct Benefit Transfer for LPG (DBT‐PAHAL) program); voluntarily limit consumptions subsidies to lower income households (the “Give It Up” program called for those who could afford LPG to eschew the subsidy); and directly targeting low income households with a subsidy to start using LPG (the PMUY) (Gould & Urpelainen, 2018). The PAHAL program alone has saved the Indian government over $8 billion up to March 2019 according to the government's figures (Press Information Bureau, 2019). PMUY has surpassed its targets and resulted in over 70 million new connections to households in just 35 months (Kar et al., 2019). Households are also able to use a loan to pay for the up‐front cost not covered by PMUY, but it is paid back by witholding the subsidy for refill cylinders until the capital is recovered. Another example of a direct incentive to promote use of LPG is the unconditional cash transfer for use of LPG to those eligible for Brazil's broader social welfare program (Bolsa Familia) (Coelho et al., 2018). Auxilio Gas is available to families earning half the minimum wage or less. However, there is mixed evidence that as an unconditional transfer as part of Bolsa Familia, households actually use it for LPG (Mazzone, 2019; Wilcox‐Moore et al., 2011). Biogas digesters have been disseminated to rural households with direct incentives. Incentives of 25% of construction or installation cost have proven to be effective. Full cost subsidies, on the contrary, may affect competitive market development (Hyman & Bailis, 2018; Zuzhang, 2013). In Indonesia, the CSR program of Nestle has provided zero‐interest loans for biogas systems (Bedi et al., 2017) to incentivize biogas use. The PMUY program in India is arguably one of the most successful direct incentive programs in terms of number of new clean cooking customers. However, such capital subsidy programs don't always result in high clean fuel usage among the rural poor. PMUY customers are low‐income by definition and their average refill rates are not as high as the prior national average (which is partially driven by richer urban and peri‐urban consumers with fewer freely accessible biomass fuel options) (Kar et al., 2019; Sharma et al., 2019).

#### 5.2.2 Reduction in Direct Disincentives

A key financial mechanism wielded by the state with direct negative impact on fuel and technology costs are taxes, including excise, VAT and duty tariffs (important for imported fuels and associated equipment). Reducing such taxes through specific policies can improve the competitiveness of clean cooking options. For example, import taxes on pelletizing equipment and on fan micro‐gasification cookstoves, which are an essential component of pellet fuel household cooking energy systems, is a major issue for private sector investors trying to introduce pellets into renewable energy markets in many countries. Policy progress in Kenya has led to biogas stoves and prefabricated digesters to be imported with tax exemption. Interviews with biogas entrepreneurs indicated that the exemption only applies to entire shipping containers and thus does not benefit small enterprises (Clemens et al., 2018). In Tanzania and Uganda, there are no tax exemptions, and the high import duties for importing biodigester equipment can limit more widespread use of biogas.

The Government of Ecuador developed policies in 2014 through *La Programa de Eficiencia Energética para la Cocción* to encourage the transition from LPG to grid‐based electricity for cooking when LPG subsidies became more expensive than the cost of electricity (Gould et al., 2018). Ecuador is in the unique position of being able to generate most, and as much as 80%, of its electricity from renewable hydropower. But when participating households received two low‐cost, single‐burner induction cooktops, cookware, and electricity for cooking through a 220V dedicated outlet and 80 kWh of electricity monthly without charge, the adoption of induction cooking was low. Households were reluctant to transition to electricity when the cost was higher than the existing LPG fuels, which had been subsidized at below market price for decades. The benefit of switching from LPG to electricity was unclear (Gould et al., 2018).

#### 5.2.3 Alteration of Market Conditions

While less common than introducing financial incentives or eliminating disincentives, government policy can also play a direct role in changing the market conditions for fuels, rather than indirectly through changing price supports. Improving the market conditions for a fuel can increase the customer base. For example, as part of the Indian government's program to expand LPG connections, plans were included for 10,000 new LPG distributors throughout India (Gould & Urpelainen, 2018). Similarly, recent moves to introduce small, 5kg LPG cylinders instead of the standard 14.2 kg cylinder in India, can influence affordability for low‐income households (although smaller cylinder means more frequent travels to points of sale). Similar government‐led efforts have enabled large‐scale adoption of LPG in other countries, including Indonesia (Thoday et al., 2018) and the majority of Latin‐American countries (Coelho & Goldemberg, 2013; Troncoso & Soares da Silva, 2017).

Market conditions can also be altered by government to discourage a fuel option. An example is kerosene for cooking that has been almost entirely replaced by LPG in the national fuel conversion program in Indonesia (Thoday et al., 2018). In Africa, removal of subsidies and favorable tax treatment of kerosene has greatly reduced its use in several African countries. Likewise, there are efforts to curtail trade in charcoal in several SSA countries in order to protect forests. These efforts have been only partially effective so far; however, the cost of evading regulations can add to the retail price of charcoal and thus reduce its competitiveness. While these policies are well motivated, they will harm households that rely on charcoal unless provisions are made to make cleaner replacement fuels available and affordable.

## 6 Framework for Integrated Policy Decision Making

A number of key decisions must be addressed by governments and decision makers to prioritize fuel choices for the residential sector in a country (e.g., tax and tariff issues, trade decisions about whether to allocate hard currency for importation of fuel, industrialization issues, environmental issues, etc.). Within any country, the mix of clean fuels for cooking, heating and lighting may be complex and is likely to consist of a mosaic of supply and use patterns. At the household level, even when users may have access to the cleanest fuel/energy options, they may continue to rely on traditional polluting fuels for certain energy tasks (stacking), either as a less expensive option or because of a personal preference for certain fuels. Consistent, affordable and accessible supply of clean fuel options is a prerequisite to limit the use of more polluting fuels.

**Figure 1:**
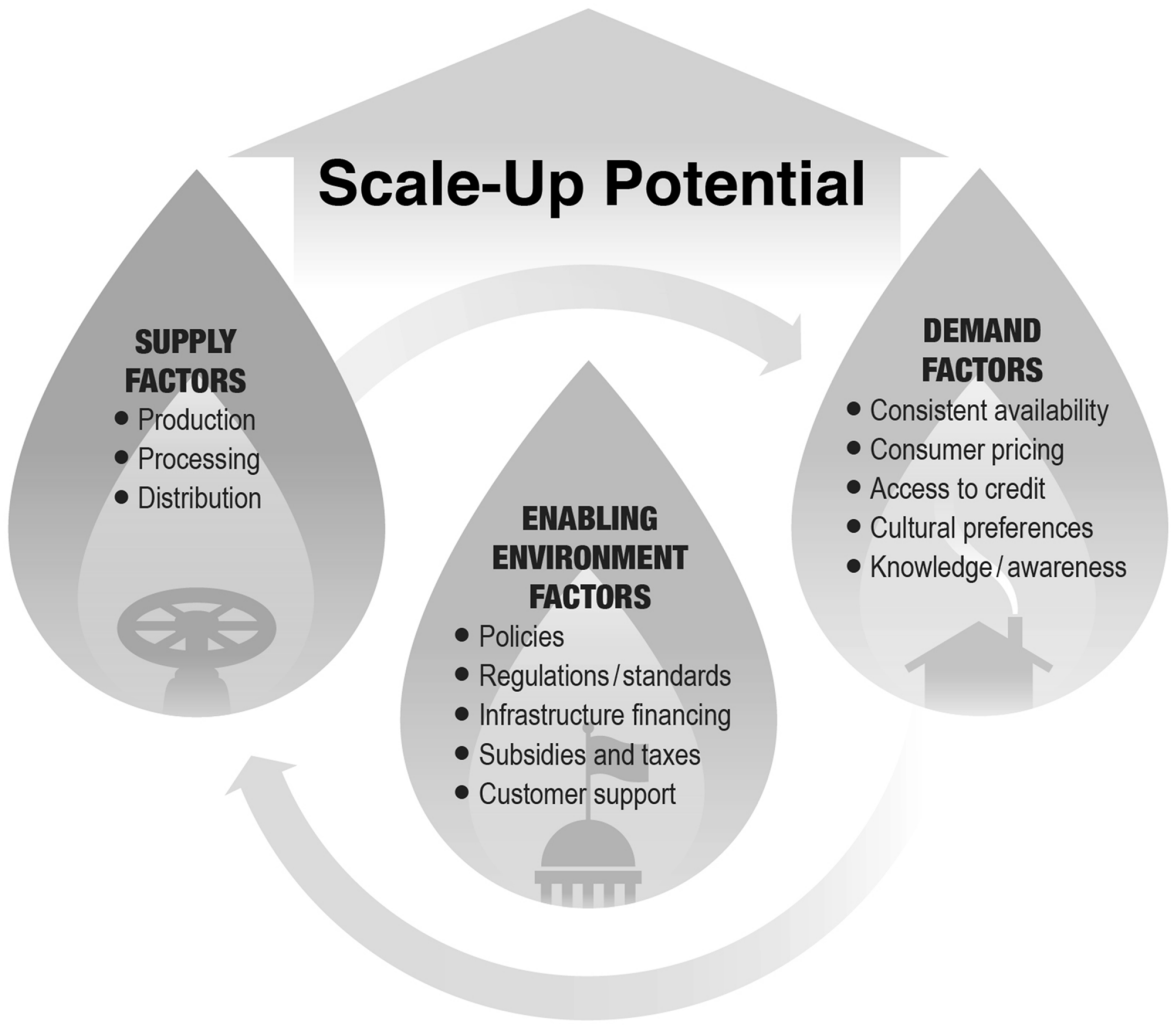
‐ Summary factors influencing scale‐up and uptake of clean household energy

National and regional scale analyses must consider existing and planned energy policies, energy prices as well as local opportunities and constraints (e.g., business opportunities, feedstock availability, storage, transport, suppliers, investors and trade partners). A simplified summary of what has been presented in sections 3‐5 is summarized in Figure 1.

Based on knowledge from various geographic settings where different clean cooking fuels have been brought to scale (Quinn et al., 2018), Table 4 summarises key aspects to consider in planning the expansion of clean fuel access. These questions are framed to initiate comparison among potential clean fuel options for a specific country considering both medium‐ and long‐term planning. Some additional considerations for decision making include:

1. Energy Considerations: Energy markets are governed by large‐scale (e.g., national, regional) and small‐scale (e.g., provincial, production) decisions. The stakeholders and decision‐makers at these different levels will be different, with those at national scales likely having more power and resources.
2. Economic Considerations: A key factor in scaling up any cooking fuel supply chain will be that the financial and business models that support the fuel supply chain result in a competitive price point or value‐for‐money to the consumer compared with alternatives as well as sufficient and lasting profit for the private businesses operating the supply chain (see Section 5).
3. Household Access and Decision Making: Clean energy has to be reliably available and accessible to households, and this objective is generally best addressed by having both private companies and public sector organizations engaged in planning and execution. At the household level, it is often the case that women do most of the cooking, as well as fuel‐gathering (e.g., biomass collection). Yet, women are not often in a position to make financial decisions about household energy technology (Austin & Mejia, 2017; WHO, 2016). Moreover, in many socio‐cultural settings, women's mobility is highly prescribed or constrained (Uteng, 2012), which further impacts their access to energy sources, and burden incurred from bringing cooking fuels all the way to the home for use. This dynamic must be considered during policy development and program implementation to ensure gender‐related barriers are mitigated so that access to household energy is equitable.
4. Health, Environment and Climate Considerations: Since one of the key objectives of clean energy is to achieve improved health with consequent gender, environment and climate co‐benefits, all household energy needs should be transitioned to clean sources, including cooking, lighting, and heating. Even when the supply of clean energy is attained, households will continue to stack and use other fuels for some aspects of household needs (Quinn et al., 2018). While addressing fuel supply issues will not fully mitigate fuel stacking with polluting fuels, it is clear that stacking provides a buffer against uncertain supply, whether for logistical, cost or availability reasons. Recognizing this, and the variability of supply chains in many countries, the use multiple clean fuels and associated technology should also be encouraged in order to minimize HAP exposures from polluting fuels such as solid fuels or kerosene.
5. Regulations and Enforcement: Both the regulation of fuels and the regulation of appliances such as stoves are necessary to standardize quality and performance. While the regulation of petroleum fuels and electricity is well established, the regulation of alternative fuels such as alcohol fuels and biomass pellets remains to be accomplished in most LMIC countries. Good cookstoves are an essential complement to clean energy, and appliance regulation is necessary to ensure that stoves are tested and certified so that consumers know which stoves are safe, functional, clean and efficient. All countries should have access to testing and certification centers. A good testing center will help to promote innovation and investment in high quality products.
6. Equity Considerations: Equity analyses help to reduce the “implementation gap,” or the difference between what policymakers plan to do and what target populations actually experience. This implementation gap is well known in the health sector, and UNICEF and others have supported the development of equity analysis tools (e.g. EQUIST), to ensure that equity is part of the system assessment (Carrera et al., 2012). The use of a equity analysis tool could be appropriate for the household energy sector too. Gaps in equity often emerge along gender boundaries, and tools such as EQUIST may highlight important socio‐cultural barriers, including constraints on how women are involved in household energy decision‐making, to successful distribution of clean energy and fuel supplies all the way into individual homes.

**Table 4:** ‐ Key questions and criteria for sustainable clean household fuel scale‐up

1. Analysis of initial conditions of the energy market	2. Economics of providing energy to the country for the household sector
1.1What are the present and projected household energy needs of the country, and their spatial and temporal patterning, under low/medium/high growth assumptions?	2.1 What are the projected costs to set up or enhance a clean fuel supply chain (from production to distribution)?
1.2What are the present sources of household energy and what are the present and projected economics of providing such energy?	2.2 What are the expected patterns of national growth and urbanization and how might those patterns impact scaling of the fuel supply (local, regional, national)?
1.3What are the present planning, data gathering, and decision‐making capacities of the country?	2.3 Who will provide the capital to build the supply infrastructure (e.g., private, public, donor)? What returns to capital investment are expected?
1.4Is civil society aware of the energy issues (and associated health and environmental impacts) and is there demand for cleaner energy currently?	2.4 Are fuel subsidies or other finance vehicles necessary to reach low‐income populations? What pricing mechanisms are necessary for different energy sources?
1.5Is there an existing distribution system for a certain fuel, either by itself or linked to another commodity? For bio‐based fuels, what is the source of feedstock and will it be limiting?	2.5 How can this transition to clean fuels be designed to minimize vulnerability to international energy pricing volatility and over what time frame should this be considered?
**3**. **Household access and awareness, including consumer demand and decision making**	**4. Status of fuel‐related regulations and enforcement**
3.1 What is the household awareness and demand for clean cooking fuel(s)? Will demand generation or behavior change be necessary to gain adoption of new fuel(s)? What gender dynamics would determin intra‐household decision‐making on clean cooking choices?	4.1 What are the existing government policies that would establish the regulatory environment for the fuel?
3.2 What are the levels of actual and perceived affordability of household energy for cooking, lighting, and heating in urban and rural areas?	4.2 Are relevant laws and regulations for the fuel adequately enforced?
3.3 What is the knowledge of and access to financial options for households to adopt the clean fuel including both initial equipment and/or ongoing fuel costs?	4.3 Can lack of proper enforcement create a threat to sustainable market expansion? How can this threat be mitigated?
3.4 What incentives for last mile‐distribution would broaden the geographic reach?	**5. Analysis of equity issues**
3.5 What is the financial infrastructure and maturity of financial markets, and associated market interest rate for (small) loans?	5.1 What strategies will reduce equity gaps in the availability and use of the clean fuel? Will different fuels or supply strategies be promoted for different income or geographic groups?
3.6 What are secondary clean fuel choices and new technologies that are appropriate for this market and provide multiple clean fuel and cooking options for households?	5.2 What populations will have lowest access to the clean fuel, and how will these populations be specifically included?
3.7 What communication strategy will be necessary to build awareness of clean fuel adoption and use?	5.3 Would a concerted stakeholder engagement process enhance equity and policy or program design?
3.8 What distribution, finance and marketing strategies can be employed to ensure that women have an appropriate role in household choices?	5.4 How can programs and policy best ensure that the needs of women, who have the greatest stake in their design and execution, be met?

### 6.1 Strength and Limitations

This study provides an analysis of critical factors that enable supply of the major clean fuel options for cooking in LMICs. In this global analysis we have attempted to cover all of these options with a common analytical framework and in making this choice we have necessarily sacrificed much technical, social and economic detail for each fuel. Our analysis is intended as a starting point for other researchers, policymakers, and practitioners, rather than a definitive statement on all options and considerations. Our sources are dominated by case studies developed by ourselves and colleagues who are members of the Clean Cooking Implementation Science Network (Quinn et al., 2018), supplemented with published academic and gray literature drawing on extensive industry and business experience. In particular, we highlight the limitations of our analysis of electricity supply, a vast field with a much broader literature than is represented in this work. Further, because our focus has been on logistical, economic, and technical supply considerations, the interactions of these factors with the fundamentally gendered social and cultural context of cooking is lacking. In this regard, we direct readers to the rapidly growing literature on clean cooking energy demand and adoption (Lewis & Pattanayak, 2012; Puzzolo et al., 2016). Finally, because of the multi‐country nature of this paper, we have not attempted to weight factors or model them formally. However, looking forward, such efforts will be important for contextualizing and building national, regional, and local clean energy plans.

## 7 Conclusions

This study summarizes key considerations for clean cooking fuel supplies in LMICs and proposes a framework to support energy decision‐makers to plan for sustainable clean household fuel scale up and optimal fuel mix. With respect to the Logic Model presented above, we see that the first three dimensions play a key role. Industry structure and services (dimension ii) vary significantly between the fuels. In some cases, fuels can be produced and marketed locally (e.g., pellets or biogas) while in other cases there is a dependency on a global supply chain (e.g., LPG). Industry structure can also differ from country‐to‐country rather than by technology. For example, the degree of privatization in the LPG market or in the electricity market varies between countries.

At the same time, the institutional environment (dimension i) influences the viability of various supply chains both directly and indirectly (e.g., by changing relative costs through specific subsidy or tax programs). Governments can, therefore, play a key role in household decision‐making by assuring that clean energy sources are available and affordable, while also making polluting fuels less available and less affordable, as well as explaining to the public why polluting fuels are a less desirable choice to make when the cleaner choices are available.

Finally energy costs and prices (dimension iii) can ultimately determine whether a clean fuel is seen as being a good value‐for‐money proposition to households. These clean fuels can be appropriate for cooking, heating and/or lighting in households, and for other appliance uses, and may be relevant for additional needs such as commercial and institutional cooking. Choice of clean fuels for cooking may also have an impact on the choice of fuels for use in power generation and transportation.

The three dimensions within the Logic Model are, of course, not the only factors influencing the adoption and use of clean fuels. The integrity and sustainability of an entire fuel supply chain (from source to distributors, and from distributors to households) is influenced by broader context‐specific factors such as policy, trade, socio‐spatial distribution (e.g. urban‐rural), state of transport networks, physical aspects (fuel density, handling, storage) and wider economic considerations. Cross sectoral influences on fuel supply (agriculture, forestry, industry, banking, transportation, digital policy, finance policy, etc.) also need to be considered and potentially adjusted to enable sustainable and financially viable supply systems.

At the same time, socio‐cultural norms and existing fuel preferences and availability will also influence household fuel choices. This requires paying particular attention to the gendered nature of both the burden of traditional fuels and of the decision‐making processes within households and communities that can influence uptake of clean fuels. While women are often bearing a significant burden from fuelwood (e.g. collection time, health effects), they are not always included in decision‐making processes that can result in a change in cooking energy systems within their household (Uteng, 2012; WHO, 2016).

The analysis presented here is intended to inform policies and programs that will ultimately make clean and modern fuels available to households in LMICs. The most appropriate clean fuel choices should be included in national household energy plans, to help guide policy decisions to enable markets to expand and build regulatory systems for the fuels.

## Conflict of Interest

The authors declare no conflicts of interest relevant to this study.
